# The impact of multidimensional excessive social media use on academic performance: the moderating role of mindfulness

**DOI:** 10.3389/fpsyg.2025.1579509

**Published:** 2025-06-23

**Authors:** Xu Ma, Qi Liu, Wen Zhang

**Affiliations:** ^1^School of Media and Communication, Wuhan Textile University, Wuhan, China; ^2^School of Film and Media, Guangxi Arts University, Nanning, China; ^3^School of Journalism and Culture Communication, Zhongnan University of Economics and Law, Wuhan, China

**Keywords:** excessive social media use, learning burnout, academic performance, mindfulness, moderating effect

## Abstract

**Introduction:**

This study investigates the negative impact of excessive social media use among college students on learning burnout and academic performance, categorizing excessive use into three types: excessive social, excessive hedonic, and excessive cognitive. Based on the SSO (Stressor-Strain-Outcome) framework, the research examines the effects of these three types of excessive use on learning burnout and academic performance. It further explores the mediating role of learning burnout and the moderating effects of mindfulness in mitigating these negative outcomes.

**Method:**

A total of 532 valid responses were collected via an online survey using standardized self-report measures to assess excessive social media use, learning burnout, academic performance, and mindfulness. Data were analyzed using SPSS 26.0 for descriptive statistics and moderation analysis, while AMOS 26.0 was employed for structural equation modeling and mediation testing.

**Results:**

Excessive social and hedonic use significantly increased learning burnout, while excessive cognitive use reduced it. Learning burnout negatively impacted academic performance and mediated the effects of all three types of social media use. Mindfulness moderated these relationships, weakening the adverse effects of excessive social media use.

**Discussion:**

The study reveals distinct effects of different types of excessive social media use on learning burnout and academic performance, underscoring the protective role of mindfulness. These findings offer valuable insights for developing interventions to enhance academic performance among college students.

## 1 Introduction

In an era of rapidly expanding communication technologies, social media platforms have become integral to college students’ daily routines, offering convenient channels for entertainment, socializing, and information exchange (Tulin et al., 2018). A recent survey on college students’ online behavior revealed that over 80% frequently engage in networking activities, with more than half spending three or more hours on social media daily (China Youth Net, 2023). While these platforms can foster connections and a sense of community (Stathopoulou et al., 2019), growing evidence suggests that excessive or compulsive use may lead to adverse outcomes, including heightened stress (Khan et al., 2021; Chen and Xiao, 2022), poor time management (Arness and Ollis, 2023), and even increased feelings of exhaustion (Cao et al., 2024).

Among the potential risks, the adverse effects of excessive social media use on academic performance have garnered significant attention (Cao et al., 2018; Jiang, 2021). A growing body of research indicates that overreliance on these platforms can precipitate learning burnout—a psychological condition characterized by emotional exhaustion, diminished motivation, and a sense of inefficacy in academic pursuits (Schaufeli et al., 2002; Fiorilli et al., 2017). Empirical studies consistently highlight a negative correlation between social media addiction or excessive use and academic performance (Salmela-Aro et al., 2017; Cao et al., 2018; Evers et al., 2020; Jiang et al., 2024). However, prior research has largely treated social media use as a monolithic construct, overlooking the heterogeneity in students’ motivations and engagement patterns. The Uses and Gratifications Theory posits that individuals are active participants in media use, driven by specific psychological needs aimed at fulfilling various motives, including social connection, entertainment, and cognitive engagement (Katz et al., 1973; Rubin, 2002; Sundar and Limperos, 2013). According to this perspective, different psychological motives lead users to adopt distinct patterns of media use, which in turn produce varying media effects. For instance, a desire for social connection may lead to more interpersonal interaction on social media, while entertainment motives may prompt passive content consumption, and cognitive motives may result in information-seeking or academic use. These patterns of use influence individual outcomes such as emotional states and performance. The multidimensional motivations underlying social media use help us gain a deeper understanding of its differential impact on individual performance (Ali-Hassan et al., 2015). To address this limitation, this study proposes a refined framework that categorizes excessive social media use into three distinct dimensions: excessive social use, excessive hedonic use, and excessive cognitive use. By examining how each dimension uniquely contributes to learning burnout and academic performance, this study seeks to uncover the nuanced mechanisms underlying these relationships.

Meanwhile, numerous studies have explored interventions to reduce excessive social media use and improve academic performance (Walther et al., 2014; Dogan et al., 2019; Hou et al., 2019), but the role of individual differences, particularly personality traits, remains underexplored. Mindfulness, a form of self-regulation that enhances emotional management and attentional control (Baer, 2009), has emerged as a promising yet understudied factor in this context. Unlike stable personality traits such as agreeableness and extraversion, which moderate the relationship between technological stressors and adverse outcomes (Rantanen et al., 2007; Srivastava et al., 2015), mindfulness is a malleable trait that can be cultivated through practices like meditation and yoga (Lomas et al., 2017). This distinction is critical, as it suggests that mindfulness may offer a unique pathway to mitigating the perception of social media as a stressor and its subsequent impact on academic performance. Building on this insight, the study also investigates how mindfulness moderates the relationship between excessive social media use and learning burnout, providing a deeper understanding of the interplay between individual traits and digital stressors.

Based on the “stressor-strain-outcome” (SSO) model, the study categorizes excessive social media use into three dimensions: excessive social use, excessive hedonic use, and excessive cognitive use (Cao and Yu, 2019). These dimensions are treated as stressors, with learning burnout as the subjective psychological experience and academic performance as the outcome. Mindfulness serves as a moderating factor between the stressors and the subjective psychological experience. Through this framework, the study examines the complex interplay among excessive social media use, learning burnout, academic performance, and individual trait differences, offering a comprehensive perspective on how digital behaviors and personal characteristics jointly shape academic performance.

This study makes several contributions to the literature. First, by categorizing excessive social media use into social, hedonic, and cognitive dimensions, it uncovers the underlying mechanisms through which different stressors affect academic performance. Second, by exploring how mindfulness moderates the relationship between excessive social media use and learning burnout, it provides empirical evidence and practical strategies for college students to use social media more effectively and mitigate its negative impacts. Together, these insights advance our understanding of the multifaceted relationship between social media use and academic performance, offering actionable recommendations for students, educators, and policymakers.

## 2 Literature review

### 2.1 Theoretical frame

The Stressor-Strain-Outcome (SSO) model, originally developed in psychology, provides a robust framework for understanding how external stressors influence individuals’ internal psychological processes and subsequent behaviors (Koeske and Koeske, 1993). The model posits that stressors—external factors that induce stress—trigger strain, which refers to subjective psychological experiences such as perceptions, emotions, and thoughts. These strains, in turn, lead to outcomes, encompassing individuals’ psychological or behavioral responses to stress (Koeske and Koeske, 1993). Importantly, stressors exert their influence on behavioral outcomes indirectly, mediated by internal psychological experiences (Ragu-Nathan et al., 2008). This model has been effectively applied to explain the psychological and behavioral consequences of stress-related situations, including those arising from social media use (Dhir et al., 2018).

In the context of this study, the SSO framework is employed to conceptualize three types of excessive social media use as distinct stressors that lead to learning burnout (strain), which in turn affects academic performance (outcome), providing a theoretical basis for understanding the mediating role of burnout in this process.

### 2.2 Excessive social media use

This study conceptualizes excessive social media use as a stressor, defined as the disproportionate allocation of time and energy to social media platforms (Caplan and High, 2007). While academic discourse has extensively debated related constructs—such as problematic use, addictive use, compulsive use, social media addiction, and social media fatigue—excessive use is distinct in denoting a less severe but still burdensome pattern of engagement, imposing psychological strain without reaching pathological harm. Research links excessive social media use to mental health issues like anxiety, depression, and low self-esteem (Bou-Hamad, 2020; Prowse et al., 2021), with longitudinal analyses identifying potential associations between excessive use and problematic internet use disorders (Zhang et al., 2024).

Building on the rich taxonomies developed by prior scholars—who have explored social media use through lenses such as functional categories (Tuck and Thompson, 2024), behavioral modes (Verduyn et al., 2021), motivational factors (Montag and Markett, 2023), and individual differences (Meynadier et al., 2024)—this study seeks to tailor its conceptualization to the specific context of academic settings. While these frameworks offer invaluable insights into the multifaceted nature of social media engagement, our focus here is narrower: we aim to capture how students’ unique motivational orientations and behavioral purposes within educational environments manifest in excessive use.

Drawing inspiration from foundational work by Cao and Yu (2019), we adopt a classification system designed to align with the academic context’s distinct demands and stressors. Rather than seeking to replace existing taxonomies, our three-dimensional framework—excessive social use, excessive hedonic use, and excessive cognitive use—represents a context-specific adaptation, prioritizing the motivational and behavioral mechanisms most relevant to students’ academic performance. This approach acknowledges the complexity of prior classifications while zeroing in on dimensions that directly inform our inquiry into how excessive use operates as a stressor in scholarly life. While users may exhibit overlapping motivations in practice, this typology categorizes use based on discrete motivational and behavioral patterns: cognitive use emphasizes information processing, hedonic use focuses on emotional enjoyment, and social use centers on relational engagement (Yu et al., 2018). As a stressor, excessive social media use has been shown to detrimentally affect psychological states and behavioral outcomes (Yu et al., 2023), providing a framework to examine its specific impacts within academic settings.

#### 2.2.1 Excessive social use and learning burnout

Social media provides a platform where students can interact, communicate, and connect with others who share similar interests (Gikas and Grant, 2013). While collaborative learning and interaction on social media can enhance learning experiences (Al-Rahmi et al., 2018; Ansari and Khan, 2020), the need to maintain numerous social connections can lead to information overload and frequent interruptions (Maier et al., 2015; Fu et al., 2020). Students may feel compelled to maintain a constant social media presence to sustain relationships (Spies Shapiro and Margolin, 2014), which can increase media multitasking, shorten their attention spans, and reduce their cognitive capacity for academic tasks. Additionally, the fear of missing out (FOMO), by eliciting persistent anxiety over social engagement and the need to stay socially connected (Montag and Markett, 2023), undermines students’ focus on academic tasks and weakens their psychological resilience, thereby exacerbating learning burnout.

In combination, excessive social use contributes to learning burnout through multiple interrelated pathways: it induces media multitasking and distractibility, amplifies FOMO-driven anxiety, and weakens self-regulatory control over goal-directed behavior. These mechanisms collectively deplete psychological resources, creating a cumulative burden that manifests in chronic academic burnout.

*H1a*: Excessive social use is positively correlated with learning burnout.

#### 2.2.2 Excessive hedonic use and learning burnout

While social media can provide a temporary escape from academic pressures through entertaining content such as humorous videos and memes (Tafesse, 2022), excessive hedonic use can lead to dependency on such content (Lopez-Fernandez et al., 2013). The enjoyment derived from social media can create negative emotions when students must return to academic tasks (Harwood et al., 2014), and distractions from entertaining content can further impair their ability to focus, contributing to learning burnout (Allaby and Shannon, 2020).

Furthermore, excessive hedonic use overloads users with sensory stimulation, leading to psychological exhaustion and information overload, which Qin et al. (2024) identify as core components of social media fatigue. This fatigue, in turn, exacerbates learning burnout by depleting cognitive resources necessary for academic persistence and problem-solving (Zheng and Ling, 2021). Individuals with lower self-control are particularly vulnerable, as they struggle to regulate their engagement with pleasurable content, prioritizing short-term enjoyment over long-term academic goals (Qin et al., 2024). This misalignment between instant gratification-seeking and academic responsibilities disrupts time management and task prioritization, accumulating into chronic stress and burnout. Collectively, these pathways highlight how hedonic overuse undermines psychological resilience, cognitive functioning, and self-regulatory processes, thereby fostering a detrimental association with learning burnout.

*H1b*: Excessive hedonic use is positively correlated with learning burnout.

#### 2.2.3 Excessive cognitive use and learning burnout

Excessive cognitive use refers to the overinvestment of cognitive resources in information processing or learning, which easily leads to cognitive overload (Iskander, 2019). Social media offers students access to vast amounts of information, which can be both beneficial and overwhelming (Zhang et al., 2016). When students encounter more information than they can effectively process, they may experience information overload, leading to negative emotional states (Ragu-Nathan et al., 2008). Specifically, when students confront overly complex or voluminous content, the resultant cognitive strain disrupts efficient information processing, eroding psychological resources and increasing vulnerability to learning burnout (Jafari et al., 2022). The challenges intensify with the fragmented attention fostered by social media-driven multitasking. Constantly toggling between academic tasks and platform interactions disrupts deep cognitive engagement, forcing the brain to allocate energy to context-switching rather than meaningful problem-solving (Novak et al., 2023; Zhao, 2023). Additionally, the pressure to engage with content through likes, shares, and comments can create anxiety, especially when responses are delayed (Lu et al., 2011, 2016). These combined factors erode students’ psychological resilience and self-regulation, ultimately fostering the core symptoms of learning burnout—exhaustion, cynicism, and reduced efficacy.

*H1c*: Excessive cognitive use is positively correlated with learning burnout.

### 2.3 Learning burnout and academic performance

Learning burnout, characterized by emotional exhaustion, reduced motivation, and negative attitudes toward learning (Schaufeli et al., 2002), can significantly impair academic performance. Exhaustion diminishes cognitive flexibility and self-assessment of learning abilities (Villavicencio and Bernardo, 2013), while low personal achievement leads to avoidance of academic challenges (Elliot and Church, 1997). Students experiencing burnout are less likely to engage in academic tasks, resulting in poorer performance (Fiorilli et al., 2017).

*H2*: Learning burnout negatively affects academic performance.

### 2.4 The mediating role of learning burnout

The SSO model suggests that strain mediates the relationship between stressors and outcomes (Koeske and Koeske, 1993). In the context of excessive social media use, learning burnout serves as a mediator between the stressor (excessive social media use) and the outcome (academic performance). Excessive use of social media diverts students’ attention and energy, increasing emotional exhaustion and burnout symptoms, which ultimately weaken academic performance (Cao et al., 2018; Evers et al., 2020; Shi et al., 2020).

*H3a*: Learning burnout mediates the relationship between excessive social use and academic performance.

*H3b*: Learning burnout mediates the relationship between excessive hedonic use and academic performance.

*H3c*: Learning burnout mediates the relationship between excessive cognitive use and academic performance.

### 2.5 The moderating role of mindfulness

Although excessive social media use can lead to a range of negative behaviors, its effects are influenced by individual differences, particularly personality traits (Lee et al., 2014). Mindfulness, as a positive personality trait, plays a critical role in regulating emotions, cognition, and behavior by fostering focus and acceptance of both internal and external stimuli in the present moment (Baer, 2003; Nyklíček, 2011). According to the mindfulness stress buffering theory, mindfulness reduces the destructive appraisal of stressful events, decreases stress responses, and promotes physical and mental wellbeing (Baer, 2003; Creswell and Lindsay, 2014). As such, mindfulness may serve as a key moderating factor in mitigating the negative effects of excessive social media use, a common technological stressor.

Mindfulness, as an intrinsic psychological resource, enables students to effectively cope with and alleviate learning burnout (An et al., 2018; Zúñiga et al., 2022; Jiang et al., 2024). While state mindfulness facilitates immediate cognitive-emotional regulation during media use episodes, dispositional mindfulness reflects a more stable attentional style that supports long-term academic resilience (Brown and Ryan, 2003). Individuals with higher levels of mindfulness are better equipped to regulate their attention and focus, reducing the risk of falling into harmful social media habits (Throuvala et al., 2020). Compared to individuals with low mindfulness, those with high mindfulness exhibit more adaptive emotional regulation skills (Dundas et al., 2013) and typically experience lower levels of social anxiety (Rasmussen and Pidgeon, 2011; Hu et al., 2024). Mindfulness training enhances emotional regulation by improving attentional control (Liu et al., 2022). This heightened emotional regulation allows individuals to maintain awareness of their behaviors while using social media and approach social interactions with greater confidence and relaxation (Apaolaza et al., 2019). By reducing social anxiety, mindfulness diminishes the reliance on social media for social validation, thereby avoiding the psychological fatigue and learning burnout caused by frequent social interactions.

In the context of excessive hedonic use, mindfulness can mitigate the overactivation of psychological and physiological states often triggered by indulging in stimulating entertainment activities, such as short videos (Moqbel et al., 2023). Individuals with higher mindfulness levels possess stronger emotional regulation abilities, enabling them to reduce distractions caused by hedonic behaviors and return more easily to learning tasks. This reduces the learning burnout associated with excessive hedonic use.

Similarly, in terms of information processing, mindfulness helps individuals consciously filter task-related information, enhancing their ability to discern the contextual appropriateness of digital information and reducing information overload (Wolf et al., 2011). Consequently, individuals with higher mindfulness are better able to identify relevant information and ignore redundant content, thereby alleviating the burnout caused by excessive cognitive use of social media.

*H4a*: Mindfulness moderates the relationship between excessive social use and learning burnout.

*H4b*: Mindfulness moderates the relationship between excessive hedonic use and learning burnout.

*H4c*: Mindfulness moderates the relationship between excessive cognitive use and learning burnout.

**Figure 1:**
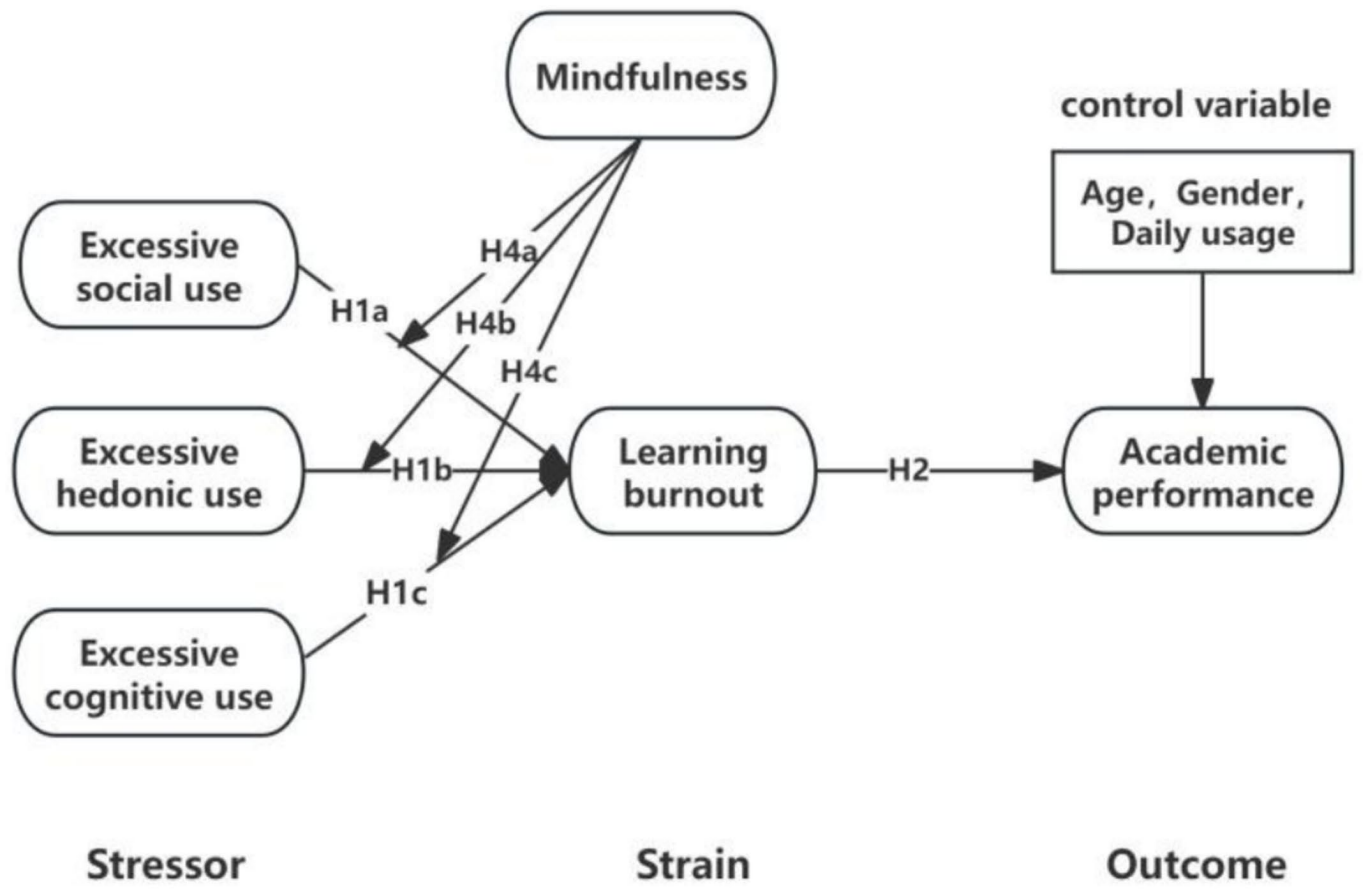
Hypothesized research model based on SSO framework.

The research model of this study is presented in Figure 1.

## 3 Questionnaire design and data collection

### 3.1 Variable measurement

Excessive social media use was measured using a scale adapted from Sun and Chao (2024), which includes three dimensions: excessive social use, evaluated by 4 items (Cronbach’s *α* = 0.72), excessive hedonic use, assessed by 3 items (Cronbach’s *α* = 0.84), and excessive cognitive use, measured by 4 items (Cronbach’s *α* = 0.90), consisting of 11 items. The items were scored using a five-point Likert scale, where 1–5 represent “strongly disagree” to “strongly agree.” Higher scores indicate a higher level of excessive social media use.

Learning burnout was measured using a scale developed by Lian et al. (2005) for Chinese college students, which includes three dimensions: exhaustion, improper behavior, and low achievement, comprising 20 items in total (Cronbach’s *α* = 0.90). All items were scored using a five-point Likert scale, with 1–5 representing “strongly disagree” to “strongly agree.” Higher scores indicate a higher level of burnout, with eight items reverse-coded.

Academic performance in previous studies is typically measured using students’ GPA. However, due to differences in the methods of GPA calculation and measurement standards among different universities in China, it is difficult to standardize GPA for comparison. Therefore, this study uses the academic performance scale developed by Yu et al. (2010) to collect self-reported academic performance from college students, which includes 4 items (Cronbach’s *α* = 0.87).

Mindfulness was measured using the Mindful Attention Awareness Scale (MAAS) developed by Brown and Ryan (2003). This one-dimensional scale consists of 15 items (Cronbach’s *α* = 0.89), with response options ranging from “1” (almost always) to “6” (almost never).

After conducting a preliminary survey, the scales were appropriately modified based on the factor loadings. Specifically, one item was removed from the excessive social media use scale, four items from the learning burnout scale, and three items from the mindfulness scale.

### 3.2 Sample and data collection

In the formal survey, we administered an online questionnaire via the Wenjuanxing platform, employing a combination of convenience sampling and stratified cluster sampling to ensure sample representativeness. Wenjuanxing is a widely used online survey tool in China that facilitates questionnaire design, distribution, and data collection. Its built-in data validation and real-time monitoring features help ensure data quality and completeness. The study used cluster sampling, dividing China into the eastern, central, and western regions according to the standards of National Bureau of Statistics of China. Two to three universities were selected from each administrative region. Teachers or students from the selected universities were contacted, and based on the actual situation, the questionnaires were distributed via WeChat groups for university students. Efforts were made to ensure that students from different grades and disciplines were included in the sample.

Regarding the minimum sample size, this study followed the common rule of thumb that suggests the sample size should be at least 10 times the number of items on the measurement scales (see, e.g., Nunnally, 1978; Tabachnick and Fidell, 2019). After modifications, the scale was comprised of 42 items; hence, the minimum required sample size was 42 × 10 = 420 responses. Data collection occurred from October 11, 2024, to November 5, 2024. A total of 675 questionnaires were collected, and after excluding responses with excessively short completion times or identical answers across all items, 532 valid responses remained, resulting in a valid response rate of 78.8%. This sample size not only far exceeds the minimum requirement but also ensures sufficient statistical power for subsequent analyses and model estimation. The demographic characteristics of the valid sample are presented in Table 1.

**Table 1:** Demographic statistics.

Item	Category	Frequency	Percentage (%)
Gender	Male	253	47.6
	Female	279	52.4
Age	17 and below	9	1.7
	18–19 years	237	44.5
	20–21 years	206	38.7
	22–23 years	67	12.6
	24 and above	13	2.4
Grade	Freshman	144	27.1
	Sophomore	173	32.5
	Junior	120	22.6
	Senior	95	17.9
Years of Use	Less than 2 years	14	2.6
	2–4 years	105	19.7
	More than 4 years	413	77.6
Daily Usage	Less than 1 h	18	3.4
	1–2 h	78	14.7
	2–3 h	131	24.6
	3–4 h	116	21.8
	More than 4 h	189	35.5

## 4 Data analysis and results

### 4.1 Common method bias test

In this study, the Harman single-factor test was used to examine common method bias. The results indicated that there are seven factors with eigenvalues greater than 1, and the first principal factor explains 26.16% of the variance, which is below the 40% threshold (Podsakoff et al., 2003). Based on this, we can conclude that there is no serious common method bias in this study.

### 4.2 Reliability and validity analysis

Reliability was tested using Cronbach’s *α* coefficient, with a value above 0.7 generally considered acceptable, and values above 0.8 regarded as excellent. The Cronbach’s *α* coefficients for excessive social media use (ESU), excessive hedonic use (EHU), excessive cognitive use (ECU), learning burnout (BO), mindfulness (MF), and academic performance (AP) were 0.72, 0.84, 0.90, 0.90, 0.89, and 0.87, respectively, all exceeding the acceptable reliability threshold of 0.7, indicating good internal consistency among the items in the questionnaire and excellent reliability of the sample data obtained through the measurement scales.

Since the measurement items were adapted from established scales, content validity is considered satisfactory. Therefore, the validity of the variables was examined from both convergent and discriminant validity perspectives. Convergent validity was tested using factor loadings, composite reliability (CR), and average variance extracted (AVE). As shown in Table 2, all factor loadings exceed 0.5. The CR values for the latent variables were 0.73, 0.90, 0.84, 0.90, 0.87, and 0.89, all exceeding the minimum standard of 0.7 proposed by Hair et al. (2019). In an ideal scenario, the AVE value should be greater than 0.5. Although the AVE value for excessive social media use was 0.47 and for mindfulness was 0.41, according to Fornell and Larcker (1981), if the AVE value is below 0.5 but the CR value for the same dimension exceeds 0.6, the convergent validity for that dimension remains acceptable. Thus, the convergent validity of the model was confirmed through these tests.

**Table 2:** Results of Descriptive Statistics, Reliability and Confirmatory Factor Analysis.

Latent variable	Observed variable	Standardized factor loading	Mean	SD	Cronbach’s *α*	AVE	C.R
Excessive social use	ESU1	0.75	3.13	0.82	0.72	0.47	0.73
	ESU2	0.55	2.67				
	ESU3	0.74	3.14				
Excessive hedonic use	EHU1	0.87	3.54	0.92	0.84	0.75	0.90
	EHU2	0.86	3.66				
	EHU3	0.86	3.62				
Excessive cognitive use	ECU1	0.75	3.28	0.85	0.90	0.56	0.84
	ECU2	0.73	2.86				
	ECU3	0.83	3.02				
	ECU4	0.68	3.11				
Learning burnout	Emotional Exhaustion	0.91	3.09	0.65	0.90	0.75	0.90
	Improper Behavior	0.90	2.97				
	Low Achievement	0.79	2.76				
Academic performance	AP1	0.81	3.40	0.79	0.87	0.63	0.87
	AP2	0.78	3.59				
	AP3	0.78	3.34				
	AP4	0.80	3.10				
Mindfulness	MF1	0.56	4.06	0.80	0.89	0.41	0.89
	MF2	0.59	4.26				
	MF3	0.68	3.96				
	MF4	0.53	3.98				
	MF5	0.68	3.87				
	MF6	0.69	3.96				
	MF7	0.71	3.78				
	MF8	0.71	4.16				
	MF9	0.60	4.43				
	MF10	0.60	3.56				
	MF11	0.75	3.82				
	MF12	0.59	4.70				

The Heterotrait-Monotrait ratio (HTMT) is a more advanced method proposed by Henseler et al. (2015) for testing discriminant validity, particularly in the context of structural equation modeling. An HTMT value below 0.85 is considered to indicate good discriminant validity between two variables. As shown in Table 3, all the HTMT values for the variables are below 0.85, confirming that the model exhibits adequate discriminant validity.

**Table 3:** HTMT discriminant validity test results.

	Excessive social use	Excessive hedonic use	Excessive cognitive use	Learning burnout	Academic performance	Mindfulness
Excessive social use	–					
Excessive hedonic use	0.57	–				
Excessive cognitive use	0.77	0.51	–			
Learning burnout	0.29	0.44	0.16	–		
Academic performance	0.14	0.19	0.05	0.66	–	
Mindfulness	0.21	0.19	0.14	0.56	0.44	–

### 4.3 Structural equation modeling

**Figure 2:**
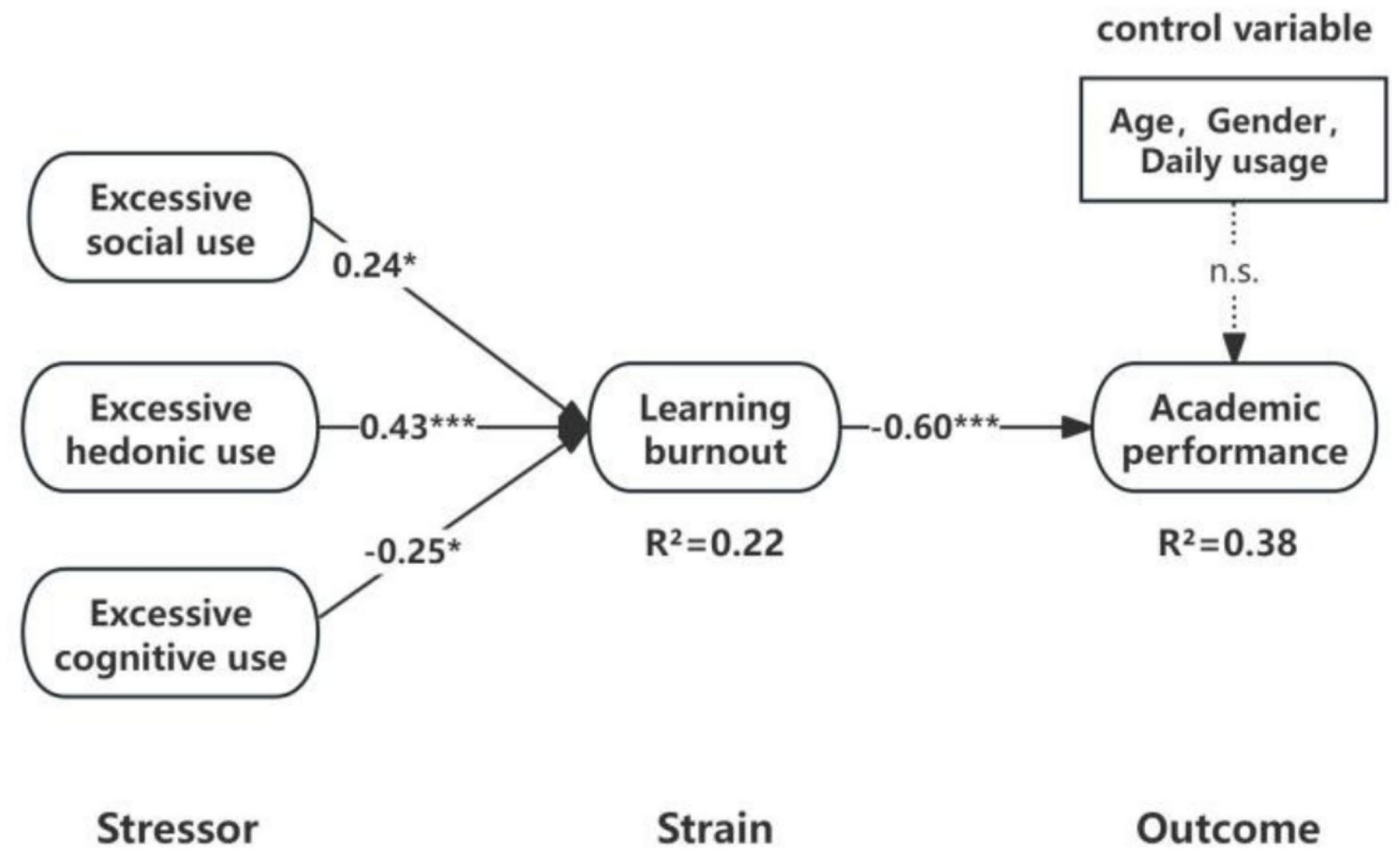
Results of model test.

This study utilized Amos 26.0 to develop a structural equation model (SEM) to investigate the impact of excessive social media use on learning burnout and the effect of learning burnout on academic performance. The results demonstrated an adequate model fit, as indicated by the following fit indices: *χ*^2^/df = 2.415, AGFI = 0.854, RMSEA = 0.052, IFI = 0.913, TLI = 0.903, and CFI = 0.912. As illustrated in Figure 2, excessive social use (*β* = 0.24, *p* < 0.05) and excessive hedonic use (*β* = 0.43, *p* < 0.001) exhibited a significant positive effect on learning burnout, supporting hypotheses H1a and H1b. However, excessive cognitive use (*β* = −0.25, *p* < 0.05) demonstrated a significant negative effect on learning burnout, failing to support hypothesis H1c. Moreover, learning burnout (*β* = −0.60, *p* < 0.001) had a significant negative impact on academic performance, thereby supporting hypothesis H2. Overall, the model accounted for 22% of the variance in learning burnout and 38% of academic performance, suggesting strong predictive validity.

### 4.4 Mediation effect test

This study employed Amos 26.0 to test the mediation effects using the Bootstrap method, with 5,000 Bootstrap samples generated through repeated random sampling. A 95% confidence interval was established. After including the mediator variable, the results are presented in Table 4 below. The mediation path “Excessive Social Media Use → Learning Burnout → Academic Performance” was found to be significant, with the 95% confidence interval excluding zero, thus supporting hypothesis H3a. Similarly, the mediation path “Excessive Hedonic Use → Learning Burnout → Academic Performance” was significant, with the 95% confidence interval not including zero, thus supporting hypothesis H3b. The mediation path “Excessive Cognitive Use → Learning Burnout → Academic Performance” was also significant, with the 95% confidence interval excluding zero, thereby supporting hypothesis H3c.

**Table 4:** Mediation path effects with confidence intervals and significance tests.

Mediation path	Effect value	S.E	Bias-corrected 95%CI			Percentile 95%CI		
			Lower	Upper	*p*	Lower	Upper	*p*
Excessive social use → learning burnout → academic performance	−0.15	0.07	−0.30	−0.01	0.04	−0.30	−0.01	0.04
Excessive hedonic use → learning burnout → academic performance	−0.26	0.04	−0.35	−0.18	0.00	−0.34	−0.18	0.00
Excessive cognitive use → learning burnout → academic performance	0.15	0.07	0.03	0.29	0.02	0.03	0.29	0.01

### 4.5 Moderation effect test

**Figure 3:**
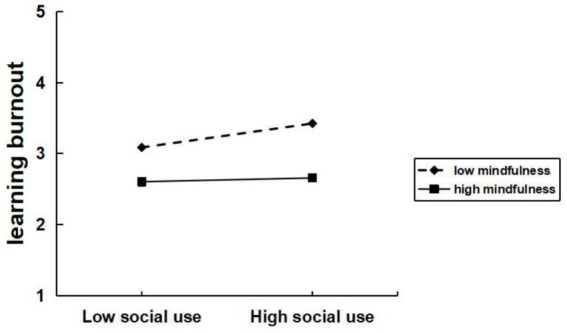
Mindfulness moderates the relationship between excessive social use and learning burnout.

**Figure 4:**
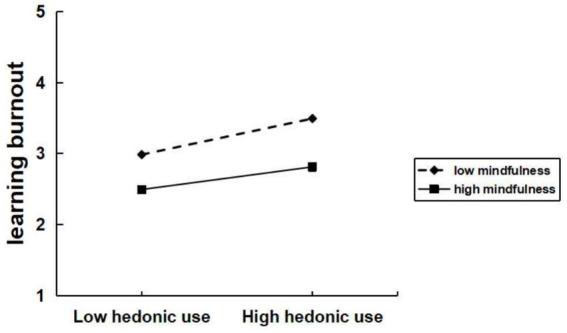
Mindfulness moderates the relationship between excessive hedonic use and learning burnout.

**Figure 5:**
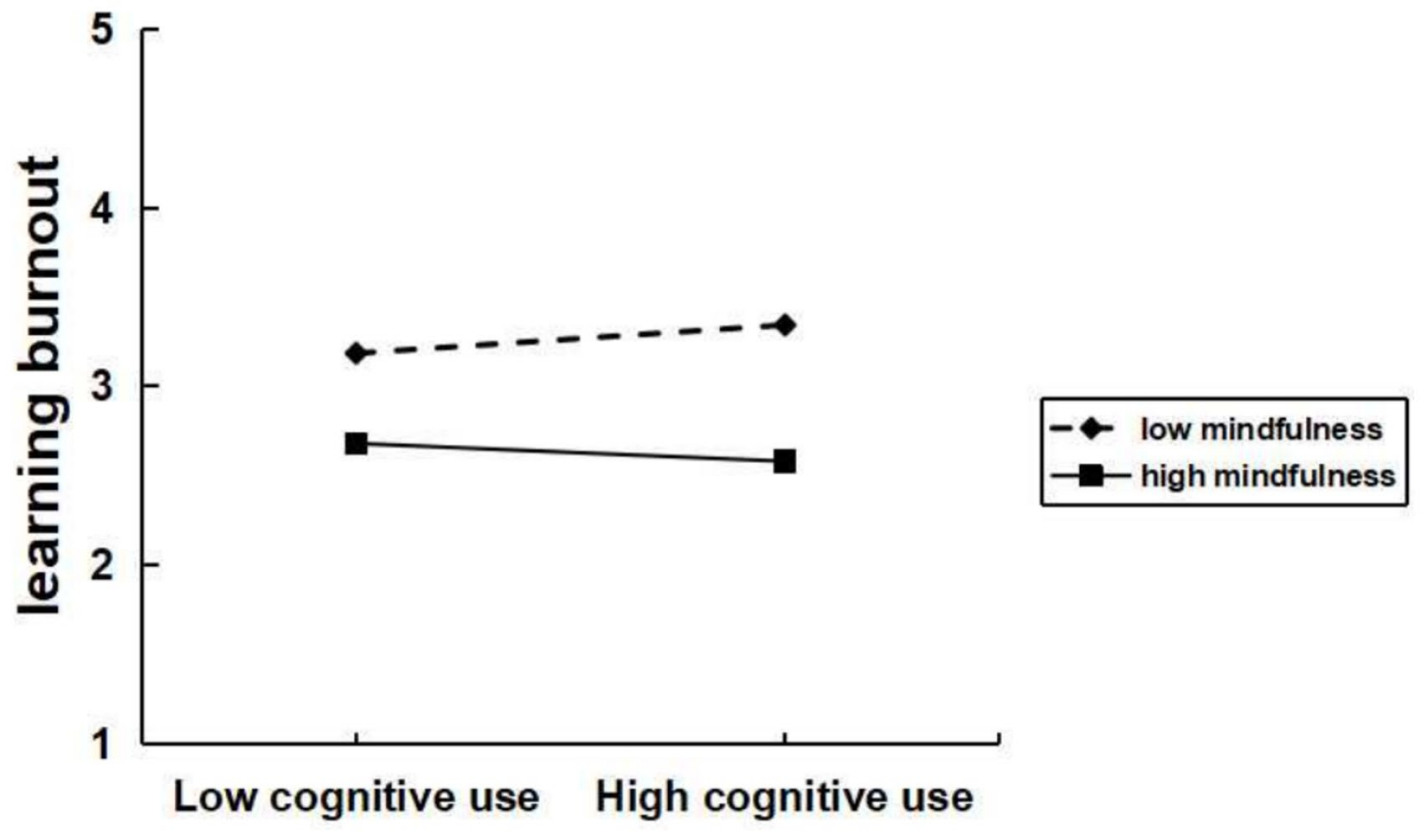
Mindfulness moderates the relationship between excessive cognitive use and learning burnout.

To test the moderation effect of mindfulness between excessive social media use and learning burnout, data analysis was performed using Model 1 of the SPSS macro program process. Based on the respondents’ mindfulness scores, they were divided into high and low mindfulness groups, and interaction plots were created with excessive social media use. As shown in Figures 3–5, significant interaction effects were found between excessive social media use and mindfulness. Specifically, for excessive social use (Figure 3), the interaction effect was significant (*β* = −0.11, *t* = −3.22, *p* < 0.05). Similarly, for excessive hedonic use (Figure 4), a significant interaction effect was observed (*β* = −0.07, *t* = −2.44, *p* < 0.05). Additionally, for excessive cognitive use (Figure 5), the interaction effect was also significant (*β* = −0.08, *t* = −2.49, *p* < 0.05). Specifically, for the low mindfulness group, the positive impact of excessive social use, hedonic use, and cognitive use on learning burnout was more pronounced. However, in the high mindfulness group, this effect was mitigated. This suggests that mindfulness plays a buffering role between excessive social media use and learning burnout. In other words, a higher level of mindfulness can reduce the learning burnout induced by excessive social media use. Mindfulness negatively moderates the relationship between excessive social use, hedonic use, cognitive use with learning burnout. Therefore, hypotheses H4a, H4b, and H4c are supported.

## 5 Discussion

### 5.1 Main findings

Excessive social media use can have significant negative effects on college students. To clarify the impact of different types of social media use on academic performance, this study employs the Stressor-Strain-Outcome (SSO) model to explore the mediating role of learning burnout and the moderating role of mindfulness in the relationship between excessive social media use and academic performance and the findings reveal several key insights.

#### 5.1.1 Differential effects of excessive social media use on learning burnout

The study found that excessive social and hedonic use of social media significantly increases learning burnout, while excessive cognitive use does not. This finding extends prior research, which has primarily focused on the positive relationship between problematic social media use and learning burnout from a unidimensional perspective (Salmela-Aro et al., 2017; Evers et al., 2020). Our results highlight that the impact of social media use on learning burnout varies depending on the purpose of use.

Excessive social use often leads to social anxiety, as individuals feel compelled to maintain relationships through frequent social media engagement (Fu et al., 2020). This creates a conflict between limited energy and social overload, ultimately contributing to learning burnout (Jiang et al., 2024). Similarly, excessive hedonic use, which involves prolonged engagement with entertainment content, can create a sense of detachment from academic pursuits. When students attempt to re-engage with their studies, they often experience burnout due to the difficulty of transitioning from leisure to academic tasks (Giunchiglia et al., 2018; Feng et al., 2019).

In contrast, excessive cognitive use of social media not only does not lead to learning burnout but also negatively predicts it. This finding contrasts with the typical assumption that excessive use of social media leads to exhaustion and disengagement. The unique nature of cognitive use diverges from social and hedonic use, which primarily focus on relational maintenance or entertainment, by prioritizing goal-directed information processing and academic engagement (Ansari and Khan, 2020; Sun and Chao, 2024).

From a Self-Determination Theory (SDT) perspective (Deci and Ryan, 1985), excessive cognitive use satisfies the fundamental psychological needs that mitigate burnout: **Autonomy:** Students actively use social media to search for academic resources, participate in MOOCs, and engage in online professional discussions (Lan and Hew, 2020). Through these activities, they exercise self-directed learning by aligning their activities with personal goals rather than external demands. This intrinsic motivation fosters a sense of control and reduces feelings of burnout (Ojo et al., 2024). **Competence:** Successfully solving academic problems or obtaining academic resources through social media like Facebook reinforces self-efficacy, which is a well-documented buffer against burnout (Valdez and Datu, 2022). **Relatedness:** Cognitively oriented use may lead students to develop a sense of connection based on shared academic goals as they exchange information and collaborate to solve problems within academic communities, thereby partially satisfying their need for relatedness and reducing feelings of isolation and learning burnout (Stoeber et al., 2011).

Furthermore, cognitive use cultivates flow experiences when the difficulty of tasks aligns with students’ capabilities (Yao et al., 2023). Engaging in activities like curating research notes or debating academic theories on professional platforms promotes deep concentration and transforms cognitive effort into rewarding absorption, rather than draining labor. These flow states enhance learning efficiency and emotional resilience, directly counteracting burnout’s core symptoms: exhaustion and disengagement.

However, this beneficial effect is conditional: it occurs when cognitive use is aligned with academic objectives and supported by self-regulatory skills such as time management (Hameed et al., 2022). While excessive cognitive use may inflate perceived academic progress, creating an illusion of competence (Chao et al., 2025), its structured, goal-driven nature contrasts with the chaotic information overload that often contributes to burnout. By emphasizing the quality of engagement (i.e., depth of processing) over the mere quantity of use, cognitive use emerges as a context-specific protective factor in burnout research, underscoring the need to differentiate social media use by motivational intent. This finding underscores the importance of distinguishing between different types of social media use when evaluating their impact on academic performance.

#### 5.1.2 The dual nature of stressors in the SSO model

Unlike previous studies that applied the SSO model to examine the uniformly negative effects of social media use on performance (Cao et al., 2018; Cao and Yu, 2019; Shi et al., 2020), this study reveals that not all stressors lead to negative outcomes. While excessive social and hedonic use exacerbate learning burnout and hinder academic performance, excessive cognitive use presents a more complex dynamic.

Stressors, by nature, are neutral, and their effects depend on how they are perceived and managed (Nelson and Simmons, 2010). In the case of excessive cognitive use, the technostress associated with information overload is counterbalanced by the opportunities it provides for information integration and knowledge deepening (Van Slyke et al., 2023). This aligns with prior research suggesting that techno-eustress (positive stress) can lead to desirable academic performance, whereas techno-distress (negative stress) results in poorer performance (Zhao et al., 2023). Thus, a dual perspective is essential when evaluating the outcomes of stressors, as their effects are not uniformly detrimental.

#### 5.1.3 The moderating role of mindfulness

The study also found that mindfulness negatively moderates the relationship between excessive social media use (social, hedonic, and cognitive) and learning burnout. This finding underscores the significant role of individual differences in shaping the impact of technostress (Srivastava et al., 2015) and supports the mindfulness stress buffering theory (Creswell and Lindsay, 2014). These effects can be understood through established mindfulness mechanisms such as attention regulation, emotion regulation, and decentering (Shapiro et al., 2006; Hölzel et al., 2011; Garland et al., 2015), which together contribute to reducing the psychological strain triggered by different types of excessive media use.

Excessive social media use for social purposes is typically characterized by heightened social anxiety, fear of missing out (FoMO), and a propensity for media multitasking. Through the cultivation of non-judgmental awareness, mindfulness can attenuate emotional reactivity to social feedback, reduce anxiety and emotional exhaustion. It also helps lessen social media rumination partly by attenuating the emotional impact of upward social comparison (Gu et al., 2022), and interrupt automatic behavioral patterns, thereby facilitating more autonomous and goal-directed engagement with social media (Apaolaza et al., 2019; Zúñiga et al., 2022; Chang et al., 2023).

Excessive hedonic use, closely associated with impulsivity and attentional distraction, may also be mitigated through mindfulness (Meynadier et al., 2025); enhanced cognitive control and increased capacity for delayed gratification serve as psychological buffers against entertainment-driven stimuli, thus reducing dependency and the risk of problematic use.

Despite its goal-oriented nature, excessive cognitive use can lead to mental fatigue when prolonged information processing occurs. Entering a mindful state helps alleviate this burden by strengthening selective attention and metacognitive regulation, thus enabling students to filter irrelevant information, manage cognitive load more effectively, and sustain attentional focus for learning tasks (Wolf et al., 2011; Chen et al., 2022). Students with higher levels of dispositional mindfulness are better able to maintain academic goal clarity, reduce psychological fatigue associated with social and hedonic overuse, and mitigate the cognitive overload linked to excessive informational engagement, thereby contributing to enhanced wellbeing and improved academic outcomes.

Given the collectivist orientation and academic intensity of Chinese higher education contexts, the regulatory role of mindfulness may be particularly salient in mitigating peer-related stressors and managing media-induced academic strain.

#### 5.1.4 Mindfulness as a protective factor against learning burnout

Learning burnout is often driven by an excessive reliance on social media and the pursuit of immediate gratification. Mindfulness training cultivates a non-judgmental, accepting attitude that not only alleviates burnout induced by frequent social interactions (Carlton et al., 2020) but also encourages students to focus on authentic, present-moment experiences. This reduces the risk of virtual entertainment addiction and enhances overall mental wellbeing (Creswell and Lindsay, 2014).

Although excessive cognitive engagement does not inherently cause learning burnout, its negative effects are more pronounced among students with low levels of mindfulness. In contrast, students with high mindfulness are better equipped to manage cognitive demands, thanks to the improved cognitive flexibility fostered by mindfulness practices (Baer, 2003). This highlights the protective role of mindfulness in mitigating the adverse effects of excessive social media use.

The findings of this study offer several practical implications for educators, policymakers, and students. First, they emphasize the importance of distinguishing between types of excessive social media use when designing interventions for learning burnout. While excessive social and hedonic use may require time management and emotional regulation strategies, excessive cognitive use—when appropriately aligned with academic goals—can be leveraged as a productive learning tool. Second, the study highlights mindfulness as a promising intervention to buffer the adverse effects of excessive media use. Integrating mindfulness into educational curricula can help students cultivate attentional control, emotional resilience, and stress management skills. For instance, mindfulness-based practices such as breathing exercises, body scans, and reflective journaling can be incorporated into mental health or physical education courses. Additionally, incorporating loving-kindness meditation can further enhance students’ spirituality and subjective wellbeing by fostering positive emotions like compassion, acceptance, and connection (Liu et al., 2020). This practice promotes an open, benevolent mindset that can reduce negative emotions and social anxiety, creating a supportive environment conducive to both mental health and academic success.

To enhance cultural relevance and acceptance among Chinese university students, mindfulness interventions can be adapted using concepts rooted in traditional Chinese philosophy—such as xiushen yangxing (cultivating the self, from Confucianism) or qingjing wuwei (tranquility and non-action, from Daoism). For example, mindfulness calligraphy or traditional art-based practices can be integrated into group sessions to deepen engagement and increase cultural resonance. Given Chinese students’ collectivist orientation and preference for group cohesion, group-based mindfulness interventions may be more effective than individual approaches. Such formats provide opportunities for peer support and shared reflection, which align with students’ relational values (Qian et al., 2022). To reduce stigma associated with mental health interventions, mindfulness can be framed positively—as a skill for emotional balance and self-improvement—rather than as a treatment for psychological problems.

Additionally, interventions should be responsive to students’ contextual stressors, including academic pressure and employment uncertainty. Mindfulness training can help students improve concentration, regulate performance-related anxiety, and clarify personal values and interests, thus enhancing their confidence and capacity to cope with uncertainty about the future. Non-clinical, creative formats—such as mandala coloring, expressive writing, or yoga-infused mindfulness—may further support engagement (Chen et al., 2024).

### 5.2 Limitations and future research

Firstly, this study is based on cross-sectional data. While it reveals the association between excessive social media use and academic performance, it cannot effectively test whether the variables are causally related. Due to the lack of longitudinal data, the study does not observe how the variables change over time and cannot clarify the direction of causality. Future research could utilize longitudinal designs or experimental methods to explore the causal relationships between variables, further validating the findings of this study. Secondly, this study did not classify social media based on their functional types or operating modes. Previous research has shown that social media with different functional modalities exert varying effects on students’ academic self-efficacy (McNallie et al., 2020). Future research could classify social media by functional type to elucidate the distinct impact pathways.

Thirdly, the sample of this study consisted exclusively of Chinese university students. Given that China, as a typically collectivistic society, may differ markedly from other cultures in terms of cultural values, educational systems, and digital communication practices (Jackson and Wang, 2013). The conclusions of this study may have strong explanatory power within the context of East Asia, characterized by collectivism, high educational pressure, and strong family ties (Choi and Nieminen, 2013). In this context, collectivist values emphasize social harmony and group reputation, which can unintentionally increase concern over others’ judgments, intensifying online social anxiety and passive behaviors like lurking (Hu et al., 2024). However, caution should be exercised in generalizing these findings to individualistic cultures, where educational systems and social dynamics differ. This is because the primary variables examined in this study may vary in their emphasis across different cultural contexts. For example, in the context of excessive social media use, studies in Asia have found that social media is often used to strengthen family and community connections, whereas in Western cultures, social media is more frequently used for personal expression and development (Alsaleh et al., 2019).

Regarding learning burnout, educational systems, societal expectations, and the availability of educational resources in different cultural contexts influence students’ experiences of academic stress and burnout. For instance, the key characteristics and measurements of academic burnout among Lebanese college students (Bou-Hamad, 2020) differ from those among Chinese students, highlighting the impact of cultural environments on academic pressure and burnout experiences. Cultural environments shape students’ learning behaviors and academic performance through varying social support systems, educational methods, and parental involvement. These effects are evident in the distinct perceptions and use of social networking services (SNSs) in different countries (Ozer et al., 2014). For example, students in the United States and Europe perceive SNSs differently in terms of their impact on academic performance. Most students do not see SNSs as having a positive or negative effect on their studies, but a larger proportion of European students believe that SNSs positively impact academic performance. They use SNSs mainly for learning-related communication and report less interference with their studies, leading to better academic outcomes. In contrast, U.S. students tend to emphasize the negative impacts of SNSs, such as distractions and procrastination, despite self-reported good time management skills. This disconnection between self-perception and actual behavior highlights how cultural differences in the perception of SNSs influence their use and academic outcomes. Research in countries like Ghana and Saudi Arabia also supports the notion of cultural differences (Alwagait et al., 2015; Boahene et al., 2019). Future research could examine the relationships among excessive social media use, academic performance, and mindfulness across diverse cultural contexts to better understand how cultural factors influence these dynamics.

## Data Availability

The original contributions presented in the study are included in the article/Supplementary material, further inquiries can be directed to the corresponding author.
